# Lithium in Tap Water and Suicide Mortality in Japan

**DOI:** 10.3390/ijerph10116044

**Published:** 2013-11-12

**Authors:** Norio Sugawara, Norio Yasui-Furukori, Nobuyoshi Ishii, Noboru Iwata, Takeshi Terao

**Affiliations:** 1 Department of Neuropsychiatry, Hirosaki University School of Medicine, 5 Zaifucho, Hirosaki, Aomori 036-8562, Japan; E-Mail: yasufuru@cc.hirosaki-u.ac.jp; 2 Department of Neuropsychiatry, Oita University Faculty of Medicine, Idaigaoka 1-1, Hasama-machi, Yufu, Oita 879-5593, Japan; E-Mails: nobuy@oita-u.ac.jp (N.I.); terao@oita-u.ac.jp (T.T.); 3 Department of Clinical Psychology, Hiroshima International University, 555-36 Kurose-Gakuendai, Higashi-Hiroshima, Hiroshima 739-2695, Japan; E-Mail: iwatan@he.hirokoku-u.ac.jp

**Keywords:** lithium, suicide rate, Japan

## Abstract

Lithium has been used as a mood-stabilizing drug in people with mood disorders. Previous studies have shown that natural levels of lithium in drinking water may protect against suicide. This study evaluated the association between lithium levels in tap water and the suicide standardized mortality ratio (SMR) in 40 municipalities of Aomori prefecture, which has the highest levels of suicide mortality rate in Japan. Lithium levels in the tap water supplies of each municipality were measured using inductively coupled plasma-mass spectrometry. After adjusting for confounders, a statistical trend toward significance was found for the relationship between lithium levels and the average SMR among females. These findings indicate that natural levels of lithium in drinking water might have a protective effect on the risk of suicide among females. Future research is warranted to confirm this association.

## 1. Introduction

Lithium is a mood-stabilizing drug that has been used effectively in the treatment of bipolar affective disorder for many years, and increasing evidence suggests its effectiveness in reducing the risk of suicide. At so-called therapeutic levels of lithium, several meta-analyses have shown anti-suicidal effects in people with mood disorders, namely, major depression and bipolar disorder [1,2,3]. In addition, a study with a randomized placebo-controlled design showed mood-stabilizing effects for low-dose lithium supplementation (400 μg/day) in former drug users [4].

Growing evidence from ecological studies suggests that natural levels of lithium in drinking water may protect against suicide. Schrauzer and Shrestha first reported a negative association between lithium levels in tap water and suicide rates in 27 counties in Texas [5]. This association was replicated in Japan and Austria [6,7]. However, a study from eastern England that used separate measurements of lithium for 47 subdivisions found no relationship between lithium levels in tap water and suicide rates [8].

The objective of this study was to investigate the relationship between lithium levels in tap water and suicide mortality in Aomori prefecture, which has the highest levels of suicide mortality rate and the lowest lithium levels in tap water in Japan. 

## 2. Methods

In 2010, the population of Aomori prefecture was 1,373,339. Aomori prefecture has 40 cities, towns, and villages and the highest levels of suicide mortality rate in Japan. Of its 40 municipalities, Aomori city has the largest population (299,520; 21%). The populations of other centers range from 237,615 (Hachinohe city) to 1,594 (Nishimeya village). Thus, the population size varies significantly across the 40 municipalities. By considering the difference in gender and age distributions in individual municipality populations, the standardized mortality ratio (SMR) for suicide was calculated for each individual municipality. The SMR is an indirect method of adjusting the mortality rate that is defined as the number of observed deaths in an individual municipality population divided by the number of expected deaths compared with the gender- and age-matched general population. We examined government statistics on suicide in Aomori prefecture and used these statistics as the average suicide SMR for the past two years across all 40 municipalities.

Lithium levels in the tap water supplies of each municipality were measured using inductively coupled plasma-mass spectrometry at Saishunkan Reassurance and Safety Laboratory Co., Ltd. (Kumamoto, Japan). This method can measure very small amounts of lithium at a minimum level of 0.1 ppb (0.1 μg/L). Lithium levels in drinking water were measured at multiple water suppliers in the same municipality, and the mean value was calculated. The distribution of lithium levels was considerably skewed (skewness = 2.66; kurtosis = 9.40). Thus, we employed log transformation (skewness = −0.27; kurtosis = −0.33) to use parametric statistical procedures. Because of significant differences in population size across the 40 municipalities, weighted least-squares regression analysis, adjusted for the size of each population, was used to investigate the association between lithium levels in drinking water and SMRs. Model 1 included only the lithium level as the independent variable. In Model 2, the density of medical institutions per 10,000 people and the unemployment rate were added as covariates. A value of *p* < 0.05 was considered significant. The data were analyzed using PASW Statistics PC software for Windows, Version 18.0.0 (SPSS Inc., Chicago, IL, USA). This study was approved by the Ethics Committee of Hirosaki University.

## 3. Results

The lithium levels in the drinking water of 40 municipalities in Aomori prefecture ranged from 0.0–12.9 μg/L. In total, the average suicide SMR in Aomori prefecture was 123 (range 96–186) for males and 105 (range 72–152) for females. 

**Table 1:** Least squares regression model weighted for the size of each population on standardized mortality ratios (SMRs) for suicide.

	Male			Female		
	β	*t* value	*p*	β	*t* value	*p*
Model 1						
Log lithium level	0.136	0.836	0.408	−0.350	−2.275	<0.05
Model 2						
Log lithium level	0.064	0.286	0.777	−0.369	−1.738	<0.10
Medical institutions density (per 10,000 population)	−0.268	−1.240	0.229	0.127	0.619	0.542
Unemployment rate	−0.115	−0.499	0.623	0.079	0.363	0.720

Table 1 shows that the SMRs for suicide across the 40 municipalities were significantly negatively associated with lithium levels in females (beta = −0.35, *p* < 0.05) but not with lithium levels in males (beta = 0.14, *p* = 0.408). After adjusting for confounders (the number of medical institutions per 10,000 people and the unemployment rate), a statistical trend toward significance remained in females (beta = −0.37, *p* < 0.10) but not in males (beta = 0.12, *p* = 0.597).

## 4. Discussion

In this study, lithium levels were significantly negatively associated with SMRs among females across 40 municipalities. However, the model that was adjusted for the density of medical institutions and the unemployment rate attenuated this association. Furthermore, although several studies have suggested that even very low lithium levels may reduce the risk of suicide, such associations were not observed among males in either the crude or the adjusted model. 

A possible explanation for this finding is that the distribution of lithium levels may have affected our results. A study in the US found a relationship between lithium levels and suicide rates, with a wider range of lithium levels (range < 1–160 μg/L) in public water [5]. However, a study in eastern England found no association, with a narrower range and a lower maximum level of lithium (range < 1–21 μg/L) [8]. In addition, traditional gender role ideology may explain the gender difference in our results. Females tend to have lower levels of organizational commitment than their male counterparts [9]. Moreover, Japanese males are traditionally conditioned to prioritize their breadwinner role [10]. During working hours, males might work and drink tap water at different municipalities apart from residing ones. Therefore, the suicide rate among males might be affected by factors other than lithium levels.

The limitations of the present study are as follows. First, the present findings were derived from a local prefecture, and therefore, only limited generalization is possible. Second, the ecological nature of this study cannot determine a causal relationship between lithium levels in tap water and suicide mortality. Our results should thus be interpreted with caution. Third, other factors, such as psychosocial and economic factors, were not taken into consideration.

## 5. Conclusions

Our study indicates that natural levels of lithium in drinking water might have a protective effect on the risk of suicide among females. However, we cannot completely rule out an association between lithium levels and suicide mortality among males due to the limitations of this study. Future research is warranted to confirm this association.
